# Species Distribution and Antifungal Susceptibilities of *Aspergillus* Section *Terrei* Isolates in Clinical Samples from the United States and Description of *Aspergillus pseudoalabamensis* sp. nov.

**DOI:** 10.3390/pathogens12040579

**Published:** 2023-04-11

**Authors:** Connie F. Cañete-Gibas, Hoja P. Patterson, Carmita J. Sanders, James Mele, Hongxin Fan, Marjorie David, Nathan P. Wiederhold

**Affiliations:** 1 Department of Pathology and Laboratory Medicine, University of Texas Health Science Center at San Antonio, San Antonio, TX 78229, USA; 2 Fungus Testing Laboratory UT Health San Antonio, 7703 Floyd Curl Drive, San Antonio, TX 78229, USA

**Keywords:** *Aspergillus* section *Terrei*, antifungal susceptibility, *Aspergillus pseudoalabamensis*

## Abstract

*Aspergillus* section *Terrei* consists of numerous cryptic species in addition to *A. terreus sensu stricto*. The treatment of invasive infections caused by these fungi may pose a unique challenge prior to diagnosis and species identification, in that they are often clinically resistant to amphotericin B, with poor outcomes and low survival rates in patients treated with this polyene. Data on the species distributions and susceptibility profiles of isolates within section *Terrei* from the United States (U.S.) are limited. Here, we report the species distributions and susceptibility profiles for amphotericin B, isavuconazole, itraconazole, posaconazole, voriconazole, and micafungin against 278 clinical isolates of this section from institutions across the U.S. collected over a 52-month period. Species identification was performed by DNA sequence analysis and phenotypic characterization. Susceptibility testing was performed using the CLSI broth microdilution method. The majority of isolates were identified as *Aspergillus terreus sensu stricto* (69.8%), although several other cryptic species were also identified. Most were cultured from specimens collected from the respiratory tract. Posaconazole demonstrated the most potent activity of the azoles (MIC range ≤ 0.03–1 mg/L), followed by itraconazole (≤0.03–2 mg/L), voriconazole, and isavuconazole (0.125–8 mg/L for each). Amphotericin B demonstrated reduced in vitro susceptibility against this section (MIC range 0.25–8 mg/L), although this appeared to be species-dependent. A new species within this section, *A. pseudoalabamensis*, is also described. Our results, which are specific to the U.S., are similar to previous surveillance studies of the *Aspergillus* section *Terrei.*

## 1. Introduction

*Aspergillus* species are able to cause a wide range of infections, with most affecting the lungs, such as acute invasive pulmonary aspergillosis, chronic pulmonary aspergillosis, aspergillomas, and allergic bronchopulmonary aspergillosis. In addition, exacerbations of chronic obstructive pulmonary disease may also be caused by these filamentous fungi. Patients at risk include those with oncologic or hematologic malignancies; solid organ transplant recipients; those receiving immunosuppressive therapy, including prolonged courses of corticosteroids; individuals with chronic pulmonary conditions, such as those with structural changes within the lung due to tuberculosis or sarcoidosis; and those with viral pneumonia (e.g., influenza or COVID-19) who require mechanical ventilation [1,2,3,4]. In epidemiologic studies, the primary causes of invasive aspergillosis include *A. fumigatus*, *A. flavus*, *A. niger,* and *A. terreus* [5,6,7]. *A. terreus* is of unique clinical importance since it is considered to be clinically resistant to amphotericin B, with poor outcomes reported in patients who have received this polyene as antifungal therapy [8,9]. Thus, in patients who are not able to be administered azoles due to adverse effects/toxicities or due to drug–drug interactions, treatment options are limited when infections are caused by *A. terreus*. Although the incidence of invasive aspergillosis caused by this species is relatively small compared to disease caused by *A. fumigatus*, higher rates of infection have been reported at certain centers, including ones in Innsbruck, Austria, and in Houston, Texas [10,11,12,13].

*Aspergillus* section *Terrei* is composed of numerous distinct species, including *A. terreus sensu stricto*, *A. citrinoterreus*, *A. hortae*, *A. alabamensis*, and *A. neoafricanus*, among others [12,13,14]. Relatively little is known about the species distribution of this section or their antifungal susceptibility profiles in clinical isolates from United States (U.S.). Although a large, international surveillance study has been published for *Aspergillus* section *Terrei*, which included a total of 370 isolates, only 10 of these were from North America [13]. Our objective was to evaluate the species distribution and antifungal susceptibilities of a large collection of *Aspergillus* section *Terrei* clinical isolates from institutions across the U.S. over a multi-year period in order to improve our understanding of this section and determine whether there may be differences between the susceptibility profiles of different drugs. We also describe a new species within the *Aspergillus* section *Terrei*, *A. pseudoalabamensis* sp. nov., which was cultured from different patients from a specific state in the U.S.

## 2. Materials and Methods

### 2.1. Fungal Isolates

In this prospective study conducted between October 2015 and January 2020, the sequential isolates of the *Aspergillus* species received in our reference mycology laboratory (Fungus Testing Laboratory, UT Health San Antonio) for species identification or antifungal susceptibility testing were included. Serial isolates from the same patients, and those from animals, environmental sampling, and nails and ears were excluded, with the exception of those included in the description of the new species. Subcultures of all isolates were prepared onto potato flakes agar on the day of receipt prior to further testing [15].

### 2.2. Species Identification

All isolates were identified at the species level by combined DNA sequence analysis and phenotypic characteristics (i.e., colony and microscopic morphology). In preparation for DNA sequence analysis, isolates were suspended in Buffer G2 (Qiagen, Valencia, CA, USA) followed by lysis using a bead beater instrument (Precellys Evolution, Bertin Instruments, Rockville, MD, USA) and the addition of proteinase K and incubation at 56 °C [16,17]. An EZ1 DNA tissue kit and a BioRobot EZ1 instrument (Qiagen) was then used to perform DNA extraction. The partial calmodulin (*CaM*) and β-tubulin (*BenA*) genes were then sequenced using the previously published primer pairs CF1L (5-GCCGACTCTTTGACYGARGAR-3) and CF4 (5-TTTYTGCATCATRAGYTGGAC-3) and Bt2a (5-TGACCCAGCAGATGTT-3) and Bt2b (5-GTTGTTGGGAATCCACTC-3), respectively [18]. BLASTn searches of the sequences were then performed in GenBank, and results with at least a 90% query coverage were considered significant with an E-value of 0.0 at 98–100% identity.

### 2.3. Description of New Species

Further phylogenetic analysis and phenotypic workup was performed on five additional isolates, two from the same patient, that were unable to be identified at the species level. For phenotypic and morphologic observations, malt extract agar (MEA, Oxoid Limited, Hampshire, UK), creatine agar (CREA), Czapek’s yeast autolysate (CYA), and potato flake agar (PFA) were used. The isolates were inoculated at three points on each plate of each medium and incubated at 25 °C, 37 °C, and 40 °C in the dark for 7 days. Colony features were examined after 7 days of incubation and colony color were recorded following the color descriptions in the Methuen Handbook of Colour [19]. For micromorphological observations, microscopic mounts were made in lactophenol cotton blue from MEA colonies. Slide cultures were also performed, and scanning electron microscopic (SEM) images were also obtained from mounts taken from MEA colonies.

For phylogenetic analysis, cultures were grown on PFA slants and incubated at 25 °C for 7 days. DNA was extracted from the harvested mycelia following previously described methods [20]. The ITS region and parts of three gene regions, *BenA*, *CaM*, and the RNA polymerase II second largest subunit (*RPB2*) genes were amplified and sequenced as previously described using the following primer pairs: BMBC-R and NL4R (ITS), Bt2a and Bt2b (*BenA*), CF1 and CF4 (*CaM*), and 5F and 7CR (*RPB2*) [20]. Bidirectional sequencing was performed using a capillary electrophoresis system (3730 × L Genetic analyzer or 3500 × L Genetic Analyzer, Life Technologies, Carlsbad, CA, USA). The obtained sequences were manually inspected, contiguous sequences were generated, and sequence alignments were produced using the Muscle program implemented in SEQUENCHER v. 5.4.6., build 46289 (Gene Codes Corp. Ann, Arbor MI, USA) [21]. The IQ-TREE software was used to construct phylogenetic trees using maximum likelihood, and the program ModelFinder was used to find the best-fitting substitution model for each gene according to the Akaike Information Criterion (AIC). Programs to measure branch support (i.e., the approximate Bayes test (aBayes) and ultrafast bootstraps programs (BS)) were implemented [22,23,24,25,26]. The strain type of *A. fumigatus* (NRRL 163) was used as an outgroup. Single-locus analyses for *ITS*, *LSU*, *BenA*, *CaM*, and *RPB2* as well as multi-locus were performed. FigTree ver 1.4.4 (http://github.com/rambaut/figtree/, accessed on 11 November 2020) was used to visualize trees and edited using Canvas Draw 3 for MAC (v. 3.0.5, build 274). To increase the robustness of the ML topologies observed in the datasets, values of aBayes implemented in IQTREE software v2.2.0 were also shown together with the BS values [24,25].

### 2.4. In Vitro Susceptibility

Antifungal susceptibility was performed via the Clinical and Laboratory Standards Institution (CLSI) broth microdilution methods for amphotericin B, isavuconazole, itraconazole, posaconazole, voriconazole, and micafungin [27]. Stock solutions at a 100X concentration of each antifungal were prepared in DMSO, and further dilutions were made in RPMI buffered with 0.165 M MOPS (pH 7.0), 0.2% glucose, and phenol red and without bicarbonate. Final testing concentrations ranged from 0.03 to 16 mg/L for amphotericin B and the triazoles and from 0.015 to 8 mg/L for micafungin. For amphotericin B and the triazoles, minimum inhibitory concentrations (MIC) were read at 100% growth inhibition after 48 h of incubation at 35 °C, while the minimum effective concentration (MEC) for micafungin, defined as the lowest concentration that resulted in morphologic changes (e.g., short, stubby, abnormally branched hyphae), was read after 24 h of incubation at the same temperature. *Hamigera insecticola* ATCC MYA-3630 (previously identified as *Paecilomyces variotii*) was included as a quality control isolate on each day of testing.

### 2.5. Data Analysis

Species distributions and sites of culture were described using descriptive statistics. MIC/MEC ranges, MIC/MEC_50_ and MIC/MEC_90_ values (i.e., MIC/MEC values that inhibited 50% and 90% of isolates, respectively), modal MIC/MEC values, and geometric mean (GM) MIC/MEC values were determined for each antifungal. When the MIC/MEC values measured were greater than the highest concentration tested, the next highest 2X concentration was assigned for statistical comparisons. Differences in GM MIC values were assessed for significance by ANOVA with Tukey’s post hoc test for multiple comparisons and correlations between isavuconazole, posaconazole, and voriconazole MICs were assessed by Pearson’s correlation using log_2_-transformed MIC values.

## 3. Results

### 3.1. Species Distribution and Sites of Culture

**Figure 1:**
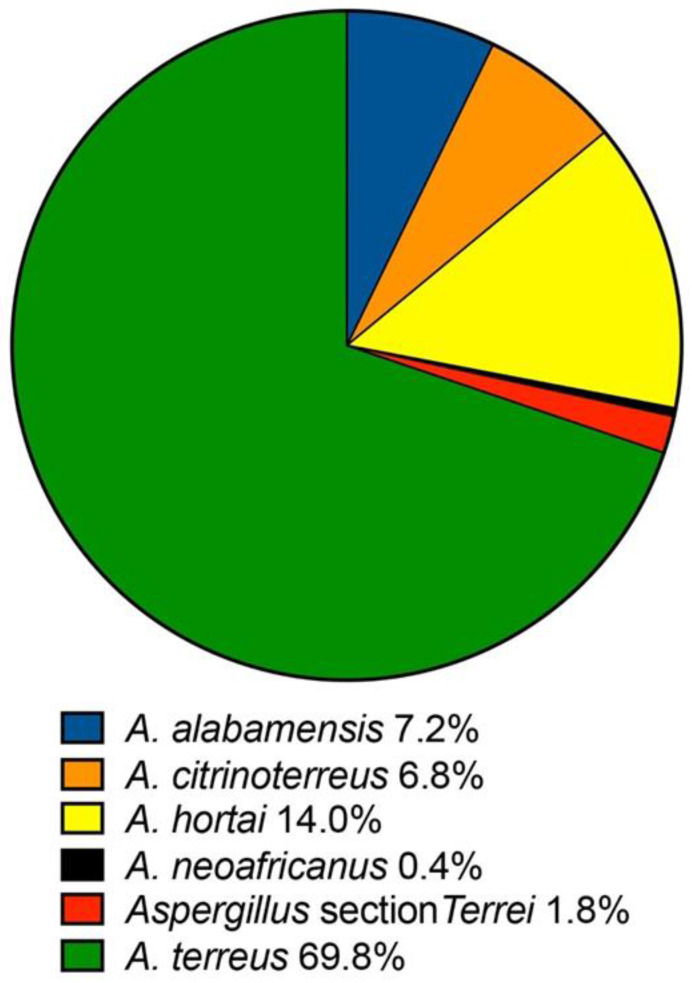
Species distribution among 278 clinical isolates within the *Aspergillus* section *Terrei*. All isolates were identified at the species level by combined DNA sequence analysis and phenotypic characteristics.

A total of 278 isolates within *Aspergillus* section *Terrei* from institutions across the U.S. were included in this analysis. Of these, *A. terreus sensu stricto* was the most prevalent species, followed by *A. hortae, A. alabamensis,* and *A. citrinoterreus* (Figure 1). The lower and upper respiratory tracts (46.8% and 22.8%, respectively) were the most prevalent sites from which the isolates were cultured (Supplemental Table S1). All other sites had less than 10% of each isolate cultured in them. Five isolates (1.8%; one *A. alabamensis*, three *A. hortae*, and one *A. terreus*) were cultured from the blood, and these may represent true infections, since members of this section are known to form aleurioconidia, or accessory conidia, that may form directly from vegetative hyphae and may be released into the bloodstream [12].

### 3.2. Antifungal Susceptibility

The MIC and MEC results for each antifungal against all isolates tested are shown in Table 1. Against section *Terrei* as a whole, the most potent in vitro activity, as measured by GM MICs, was observed with posaconazole followed by itraconazole. The activities of isavuconazole and voriconazole were similar against these isolates, with both GM MICs, modal MICs, and MIC_50_ and MIC_90_ values being the same or within one dilution of each other. Not surprisingly, amphotericin B demonstrated less in vitro activity, as evident by higher GM MIC, modal MIC, and MIC_50_ and MIC_90_ values than the triazoles. This pattern of in vitro activity also held true for each of the individual species within section *Terrei*. Interestingly, the amphotericin B GM MIC and MIC_90_ values were higher against *A. citrinoterreus* isolates (2.97 mg/L and 8 mg/L, respectively) than those of other species and were lowest against *A. hortae* isolates (0.862 mg/L and 2 mg/L, respectively). Overall, the amphotericin B and triazole MICs against *A. neoafricanus* and members of the section that were not able to be identified at the species level were similar to those observed for the other species, although three of the five isolates had amphotericin B MICs ranging from 1–8 mg/L.

CLSI has not yet established breakpoints for any antifungal against members of section *Terrei.* However, epidemiologic cut-off values (ECVs) have been established for amphotericin B and the triazoles against *A. terreus sensu stricto* [28]. Overall, most isolates were categorized as wild-type since the majority MIC values were at or below the ECVs for posaconazole (ECV 1 mg/L; 100% wild-type), voriconazole (ECV 2 mg/L; 98.86% wild-type), itraconazole (ECV 2 mg/L; 100% wild-type), and amphotericin B (ECV 4 mg/L; 99.25% wild-type). However, the number of wild-type isolates was lower for isavuconazole (ECV 1 mg/L; 93.23% wild-type), although it was still greater than 90%.

**Figure 2:**
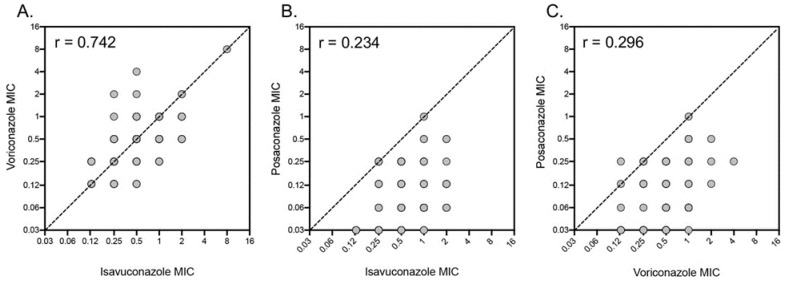
Minimum inhibitory concentration (MIC) comparisons between (**A**) isavuconazole and voriconazole (*n* = 254), (**B**) isavuconazole and posaconazole (*n* = 152), and (**C**) voriconazole and posaconazole (*n* = 150) against the *Aspergillus* section *Terrei* clinical isolates. MICs for the azoles were measured after 48 h of incubation at 35 °C as the lowest concentration that resulted in 100% inhibition of growth. Pearson’s r-values are also shown.

Direct comparisons were also made between the triazoles isavuconazole, posaconazole, and voriconazole against isolates for which all three drugs were tested against at least ten strains (Table 2). Posaconazole demonstrated significantly more potent in vitro activity, as assessed by GM MIC values (*p*-value < 0.001 for all comparisons), than that of both isavuconazole and voriconazole against *Aspergillus* section *Terrei* isolates as a whole and also against individual species. Lower modal MICs and MIC_50_ and MIC_90_ values were also observed with posaconazole. The correlations between isavuconazole and voriconazole, isavuconazole and posaconazole, and voriconazole and posaconazole MICs were also evaluated. The best correlation was observed between isavuconazole and voriconazole (Pearson’s r-value 0.742) (Figure 2). In contrast, the correlations between posaconazole and either isavuconazole or voriconazole MICs were low (Pearson’s r-values of 0.234 and 0.296, respectively).

Micafungin also demonstrated potent activity, with MEC values at the lowest concentration tested (0.015 mg/L) for the vast majority of isolates tested. This was consistent regardless of species. However, since the endpoint for the echinocandins is different than that of the triazoles and amphotericin B, comparisons between this antifungal and the other drug classes were not made.

### 3.3. Description of New Species

**Figure 3:**
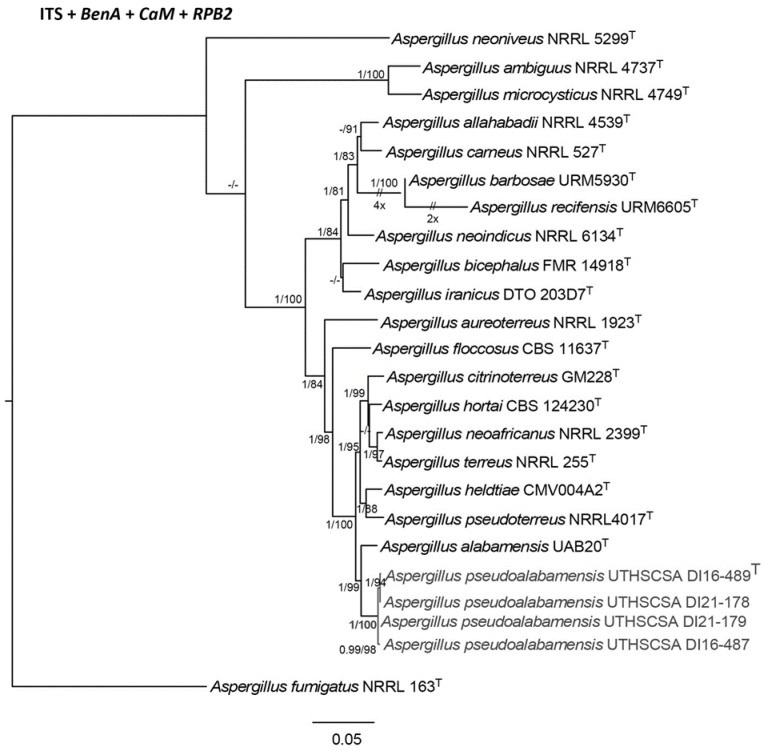
Maximum likelihood phylogeny of combined ITS, *BenA*, *CaM*, and *RPB2* showing the placement of the new species of *Aspergillus* in the section *Terrei* and its relationships with other species in the section. Support values for SH-aLRT (≥80%) and aBayes (≥0.95), BS (≥75%), respectively, are indicated on the nodes. ^T^ = represent type strains. Strain accession numbers are shown after the name. Dashes represent values lower than SH-aLRT (80%), aBayes (0.95), and BS (75%).

As previously described, five isolates underwent further phylogenetic analysis and phenotypic workup for the description of a new species, and each these isolates were cultured from four patients from three different institutions in Colorado, two in 2015 and one each in 2016, 2019, and 2020, respectively. All were presumptively identified as *Aspergillus* species belonging to the *Aspergillus* section *Terrei* based on phenotypic characteristics typical of species within this section. By BLASTn analysis of the *BenA* and *CaM* sequences, each isolate was found to be close to *A. alabamensis*, but were distant from other species within this section based on percent identity values (Supplemental Table S2). Subsequent phylogenetic analyses using additional regions (i.e., ITS and *RPB2*) were performed to study their relationship to species in section *Terrei*. Reference sequences (specifically from ex-types) of representative strains were obtained from GenBank and were included in the phylogenetic analyses [29,30,31,32]. Initially, phylogenetic analyses were performed on the datasets of individual genes (ITS, *BenA*, *CaM*, *RPB2*) to apply the genealogical concordance phylogenetic species recognition (GCPSR) concept [33]. The single-gene phylogenies showed that although the isolates were most phylogenetically close to *A. alabamensis*, they form a distinct lineage, which was confirmed by the phylogeny of the combined genes (Supplemental Figure S1 and Figure 3, respectively). The existing strains of *A. alabamensis* are diverse phylogenetically [30]. To show that our isolates are distinct, a separate *CaM* phylogenetic analysis was carried out, which included 24 additional strains of *A. alabamensis.* The resulting *CaM* phylogeny showed that our strains still constitute a distinct lineage. Here, we introduce a new species, *A. pseudoalabamensis.* The morphology of the new species is compared to *A. alabamensis*, and distinguishing characteristics are provided below.

## 4. Taxonomy

**Figure 4:**
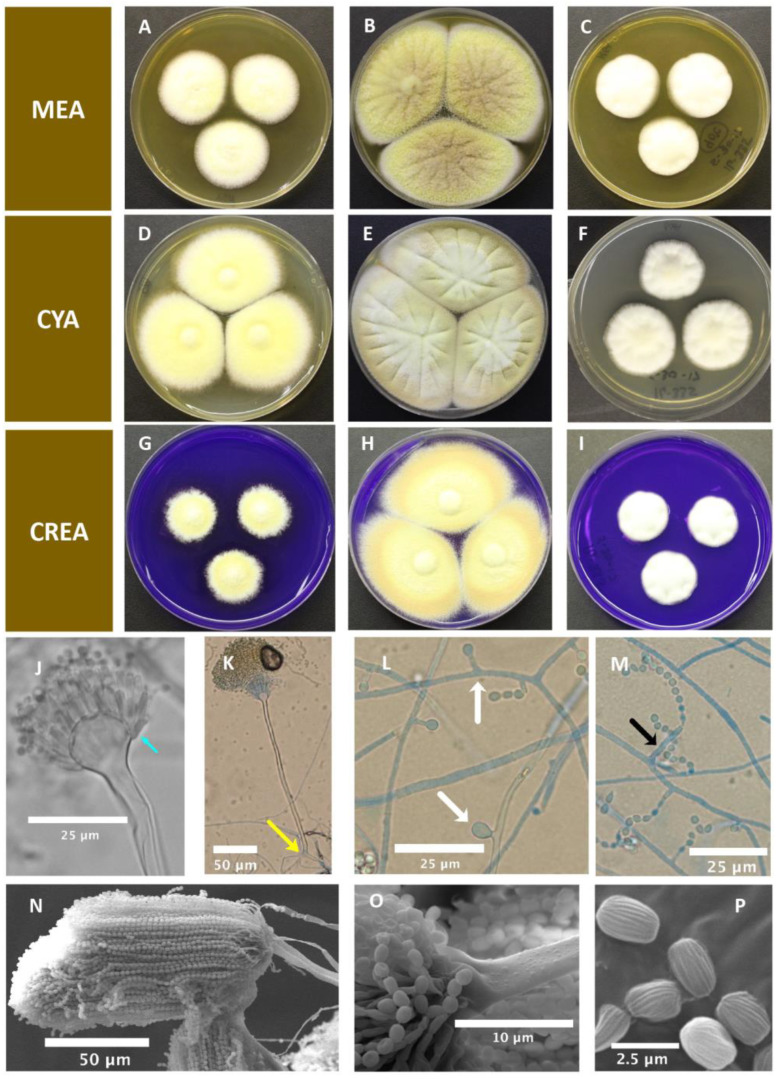
*Aspergillus pseudoalabamensis* sp. nov. (UTHSCSA DI16-489; CBS 149790). Colonies after 7 d at 25 °C, 37 °C, and 40 °C, respectively on: (**A**–**C**) MEA; (**D**–**F**) CYA; (**G**–**I**) CREA; (**J**) vesicle, (blue arrow) metulae and phialides; (**K**) (yellow arrow) foot cell; (**L**) (white arrow) accessory conidia; (**M**) (black arrow) reduced vesicle; (**N**–**P**) (SEM images)—(**N**) columnar conidial head, (**O**) finely roughened conidiophore, and (**P**) striated conidia.

*Aspergillus pseudoalabamensis* C. F. Cañete-Gibas, C. Sanders, J. Mele and N. P. Wiederhold, sp. nov. (Figure 4).

Mycobank: MB847407.

Classification—Fungi Dikarya Ascomycota Pezizomycotina Eurotiomycetes Eurotiomycetidae Eurotiales Aspergillaceae *Aspergillus*, Infragen. class: subgen. Circumdati, sect. Terrei, ser. Terrei.

Etymology: *pseudoalabamensis* refers to *A. alabamensis*, a species which is morphologically similar and phylogenetically close.

Diagnosis—The conidial color en masse is pale yellow to alabaster (2A3/ 5B2) becoming greyish to brownish orange (5B3-5C3) when mature. The stipe is finely roughened, and conidia are ellipsoidal and striated. The species does not grow at 50 °C.

*Typus*. United States of America, Colorado, tracheal aspirate from a human patient; 2016; holotype = H-25222; Ex-type = UTHSCSA DI16-489 = CBS 149790; *ITS* barcode: OL765285; alternative markers: *BenA* = OL792694; *CaM* = OL764871; *RPB2* = OL764876.

Additional materials examined. United States of America, Colorado.

Isolate from the right ethmoid sinus of a human patient, 2015, culture UTHSCSA DI16-487 = CBS 149791; ITS = OL765283; *BenA* = OL792695; *CaM* = OL764872; *RPB2* = OL764877.

Isolate from the right ethmoid sinus of a human patient, 2015, culture UTHSCSA DI16-488 = CBS 149792; ITS = OL765284; *BenA* = OL792696; *CaM* = OL764873; RPB2 = OL764878.

Isolate from an ear swab of a human patient, 2019, culture UTHSCSA DI21-178 = CBS 149793; ITS = OL765286; BenA = OL792693; *CaM* = OL764870; *RPB2* = OL764875.

Isolate from a sputum sample of a human patient, 2020, culture UTHSCSA DI21-179 = CBS 149794; *BenA* = OL792692; *CaM* = OL764869; *RPB2* = OL764874.

### 4.1. Phenotypic Characteristics

Colony morphology—Colony diameter after 7 days (mm): 25 °C—CYA 46, MEA 32, CREA 25–26; 37 °C—CYA 70, MEA 65, CREA 57–60; 40 °C—CYA 29–32, MEA 27–30, CREA 22; 50 °C—no growth on all media.

CYA, 25 °C, 7 days: colonies slightly raised at the center and radially sulcate; margins entire, regular; mycelia white; sclerotia absent; texture velvety; degree of sporulation poor at margins and sparse at the center; conidia en masse pale yellow to alabaster; soluble pigments absent; exudates absent; reverse pigmentation cream (Figure 4D–F).

MEA, 25 °C, 7 days: colonies slightly raised at the center and radially sulcate; margins entire, regular; mycelium white; sclerotia absent; texture velvety; degree of sporulation poor to moderate; conidia en masse greyish to brownish orange; soluble pigments absent; exudates absent; reverse pigmentation cream (Figure 4A–C).

CREA, 25 °C, 7 days: colonies slightly raised, margins entire, regular; mycelia white; sclerotia absent; texture velvety; degree of sporulation poor to moderate; conidia en masse greyish to brownish orange; no acid production observed (Figure 4G–I).

### 4.2. Micromorphology

Conidiophores biseriate. Stipes hyaline, 150–325 × 2.5 μm, smooth-walled to finely roughened, aseptate, foot cell mostly asymmetric. Conidial heads densely columnar; vesicles, subglobose, 12.5–22.5 μm in diameter. Metulae (L) 5 μm. Phialides (L) 5–6.5 μm, cylindrical. Conidia ellipsoidal, striated, 2–2.3 × 1.42–1.60 μm. Accessory conidia stalked. No ascomata, ascospores, or Hülle cells observed.

Notes: *A. pseudoalabamensis* is phylogenetically closely related to *A*. *alabamensis*; however, it can be differentiated from this species by *BenA*, *CaM*, and *RPB2* sequence data. The conidia of *A. pseudoalabamensis* are pale yellow to greyish to brownish orange en masse, while those of *A. alabamensis* are bright yellowish brown to cinnamon-brown [29]. Furthermore, as demonstrated by light microscopy and SEM, the conidiophores of A. *pseudoalabamensis* are shorter (150–325 × 2.5 μm) and finely roughened than those of *A. alabamensis*, which are longer (150–500 μm × 4.5–6 μm) and smooth (Figure 4J–P) [29].

## 5. Discussion

*Aspergillus terreus* is one of the four main species causing infections by the genus *Aspergillus*, although the number of infections caused by this species is relatively small compared to *A. fumigatus* [5,6,7]. However, invasive and chronic infections secondary to *A. terreus* have fewer treatment options due to poor response rates observed in patients treated with amphotericin B formulations in various studies [8,9]. In a retrospective cohort study of 83 cases of proven or probable invasive aspergillosis due to *A. terreus*, Steinbach et al. reported that patients who received primary therapy with voriconazole had a higher overall survival 64.7%) versus those who received an amphotericin B formulation as primary therapy (26.2%; *p* = 0.01) [8]. This difference in survival rate was even greater for voriconazole compared to patients who only received amphotericin B for the duration of treatment (16.7%; *p* < 0.01). Similarly, Hachem et al. conducted a retrospective chart review of patients between 1995 and 2001 at MD Anderson Cancer Center in Houston, Texas, whose cultures were positive for *Aspergillus* species [9]. In all, 32 patients with invasive aspergillosis due to *A. terreus* and 33 patients in whom the disease was caused by *A. fumigatus* were evaluated. Interestingly, response rates to therapy were low in both groups, with only 39% responding in the *A. fumigatus* group and 28% in the *A. terreus* group. In those who received treatment with amphotericin B, response rates were 29% and 16%, respectively. Susceptibility data were available for 30 isolates (16 *A. fumigatus* and 14 *A. terreus*), with amphotericin B showing reduced in vitro activity against *A. terreus* (MIC_90_ 4 mg/L) compared to *A. fumigatus* (1 mg/L).

The poor clinical outcomes associated with the amphotericin B treatment of *A. terreus* infections are often attributed to microbiologic resistance. Indeed, some studies have reported elevated amphotericin B MICs against *A. terreus* isolates. In an international, prospective multicenter survey of the prevalence of *A. terreus* species complex infections and amphotericin B susceptibility, Risslegger et al. reported amphotericin B modal and MIC_90_ values of 8 mg/L and 16 mg/L, respectively, against 370 isolates [13]. Based on their calculated amphotericin B ECV of 4 mg/L, just over half (52.4%) were classified as non-wild-types. Similar results were observed in a multicenter study conducted in France where the amphotericin B MIC_90_ was 8 mg/L [34]. However, not all in vitro studies have reported amphotericin B MICs as high as these levels. Several studies have noted greater amphotericin B in vitro susceptibility against *A. terreus* isolates, as reflected by MIC_90_ values of 2 mg/L [5,35,36,37]. The results of these later studies are consistent with our findings, as the MIC_90_ for amphotericin B against all members of section *Terrei* was 4 mg/L. However, we also saw variability with amphotericin B MIC results between the different species of this section, with the highest MIC_90_ and GM MIC against *A. citrinoterreus*. This is consistent with the results of Imbert et al., who also reported higher amphotericin B MIC_90_ and GM MIC values against this species [34]. Risslegger et al. reported higher amphotericin B MICs against all cryptic species compared to *A. terreus sensu stricto*, although this is not consistent with our results. Because of the variability in amphotericin B MICs and the lack of correlation between low values and positive clinical outcomes, CLSI now recommends against antifungal susceptibility testing of this polyene for clinical diagnostic purposes [38].

Our azole susceptibility results are in line with those reported by others, with posaconazole having the most potent in vitro activity and the MICs of voriconazole and isavuconazole closely mirroring each other, as reflected by Pearson’s r-value of 0.742. This is consistent with what our group and others have reported for voriconazole and isavuconazole against *A. fumigatus* [39,40]. As previously noted, CLSI has not yet established breakpoints for any antifungal against *A. terreus*, although ECVs are available. These ECVs were established, in part, based on MIC data provided by multiple laboratories from several different countries [41,42,43]. The MIC ranges, modal MICs, and percent of isolates at or below the ECV from these studies (amphotericin B 0.12–32 mg/L, 2 mg/L, 94.7%; isavuconazole 0.06–2 mg/L, 0.25 mg/L, 99.7%; itraconazole 0.03–1 mg/L, 0.25 mg/L, 100%; posaconazole ≤ 0.03–2 mg/L, 0.25 mg/L, 99.7%; voriconazole 0.03-≥ 4 mg/L, 0.5 mg/L, 99.1%) are very similar to the results presented here, with the exception of the isavuconazole MIC range, which increased to 8 mg/L in the current study, and the percent of isolates at or below the isavuconazole ECV, which was 94.7% here. The reasons for these differences are unknown but may warrant the continued monitoring of isavuconazole MICs against *A. terreus* isolates over time.

*Aspergillus* section *Terrei* is now recognized as consisting of at least 14 cryptic species in addition to *A. terreus sensu stricto* [14,34,44], although less than half of these have been implicated in human disease [13,29,34,45,46]. In the current study, the majority of isolates were identified as *A. terreus sensu stricto* (nearly 70%)*,* followed by the cryptic species *A. hortae, A. alabamensis,* and *A. citrinoterreus*. Similar species distributions have been reported in other large surveillance studies, where *A. terreus sensu stricto* was the predominant species cultured from humans [13,34]. As with other reports, the majority of isolates in our study were cultured from either the lower or upper respiratory tract.

The classification of *Aspergillus* species currently follows the polyphasic approach, wherein morphology, physiology, and molecular data are combined [18,47,48]. The isolates studied were obtained from the same locality (i.e., the state of Colorado, USA), from four different patients and at different times. These isolates were found to be conspecific and formed a distinct lineage within the *Aspergillus* section *Terrei*. Although there are various characteristics that can be used for the identification of section *Terrei* species, such as colony characteristics on various media and micro-morphologies, for example, the length of conidiophores, the color of conidial heads, and the production of accessory or secondary conidia, to name a few, making a distinction between species is still challenging as these features can change due to environmental conditions. Molecular data are advantageous in delimiting species in this section because these do not alter with changes in environmental conditions, unlike morphological characteristics. Based on morphological characteristics and the phylogenetic concordance of the various loci used, these isolates represent a new species named *A. pseudoalabamensis*, which is presented here. Utilizing the approach of congruent topologies derived from single genes has been found to be a good tool in species delimitation in the genus *Aspergillus* [29,30,47,48,49]. ITS is an accepted barcode for fungi, but in this study, ITS was not reliable in distinguishing the species in the section, and *BenA*, *CaM,* and *RPB2* are often used instead. Geographical distribution is not always known for many fungal species. This report, however, suggests that *A. pseudoalabamensis* is possibly endemic in Colorado. The knowledge of endemic mycosis will help in the research for disease prevention and management.

Although these results add to the literature on antifungal susceptibility and the species distribution of *Aspergillus* section *Terrei* throughout the U.S., not least due to the inclusion of the description of a new species, *A. pseudoalabamensis*, from a specific geographic area, this study is not without limitations. No clinical data were available to determine whether worse outcomes were associated with different species, sites of infection, or susceptibility profiles. In addition, we are unable to determine whether different cryptic species were associated with specific regions, as many of these isolates were received from other commercial or reference laboratories; hence, their geographic origin is unknown. Therefore, further studies are warranted.

In conclusion, our results demonstrate species diversity within the clinical isolates of *Aspergillus* section *Terrei* in the U.S., although the predominant species remains *A. terreus sensu stricto*. The azoles posaconazole, itraconazole, isavuconazole, and voriconazole have potent in vitro activity against members of this section as a whole and against the individual species. Amphotericin B demonstrated reduced susceptibility compared to the azoles, but this also varied between different species. Overall, these results from the U.S. are consistent with those of previous surveillance studies from other countries.

## Figures and Tables

**Table 1:** Minimum inhibitory concentration (MIC) and minimum effective concentration (MEC) ranges, MIC/MEC_50_ and MIC/MEC_90_ values, modal MIC/MECs, and geometric mean (GM) MIC/MEC values for amphotericin B, isavuconazole, itraconazole, posaconazole, voriconazole, and micafungin against *Aspergillus* section *Terrei* clinical isolates. MICs for amphotericin B and the azoles were measured after 48 h of incubation at 35 °C as the lowest concentration that resulted in a 100% inhibition of growth. MECs for micafungin were measured after 24 h of incubation at 35 °C as the lowest concentration that resulted in morphologic changes (i.e., short, stubby, abnormally branched hyphae). Some values were not calculated as their number of isolates tested was less than 10. All values reported as mg/L.

Species	Antifungal (No. Isolates Tested)	MIC/MEC Range	MIC/MEC_50_	MIC/MEC_90_	Modal MIC/MEC	GM MIC/MEC
*Aspergillus* section *Terrei*	Amphotericin B (195)	0.25–8	2	4	2	1.63
	Isavuconazole (276)	0.125–8	0.5	1	0.5	0.527
	Itraconazole (148)	≤0.03–2	0.25	1	0.5	0.196
	Posaconazole (152)	≤0.03–1	0.06	0.25	≤0.03	0.087
	Voriconazole (254)	0.125–8	0.5	1	0.5	0.445
	Micafungin (80)	≤0.015–0.03	≤0.015	≤0.015	≤0.015	≤0.015
*A. terreus*	Amphotericin B (134)	0.25–8	2	4	2	1.80
	Isavuconazole (192)	0.125–8	0.5	1	0.5	0.639
	Itraconazole (101)	≤0.03–1	0.25	1	1	0.236
	Posaconazole (106)	≤0.03–1	0.125	0.25	0.25	0.104
	Voriconazole (175)	0.125–8	0.5	1	0.5	0.236
	Micafungin (58)	≤0.015–0.03	≤0.015	≤0.015	≤0.015	≤0.015
*A. hortae*	Amphotericin B (28)	0.5–4	1	2	1	0.862
	Isavuconazole (39)	0.125–2	0.25	1	0.25	0.383
	Itraconazole (22)	≤0.03–0.5	0.06	0.5	≤0.03	0.081
	Posaconazole (19)	≤0.03–0.25	0.06	0.25	≤0.03	0.056
	Voriconazole (36)	0.125–1	0.25	1	0.25	0.292
	Micafungin (10)	≤0.015	≤0.015	≤0.015	≤0.015	≤0.015
*A. alabamensis*	Amphotericin B (14)	0.5–4	1	2	1	1.35
	Isavuconazole (20)	0.125–2	0.25	1	0.25	0.319
	Itraconazole (14)	≤0.03–2	0.25	1	≤0.03	0.174
	Posaconazole (9)	≤0.03–0.5	---	---	---	---
	Voriconazole (19)	0.125–2	0.25	1	0.25	0.373
	Micafungin (4)	≤0.015	---	---	---	---
*A. citrinoterreus*	Amphotericin B (14)	1–8	2	8	2	2.97
	Isavuconazole (19)	0.125–1	0.25	1	0.25	0.311
	Itraconazole (8)	≤0.03–0.5	---	---	---	---
	Posaconazole (15)	≤0.03–0.25	≤0.03	0.25	≤0.03	0.055
	Voriconazole (18)	0.125–1	0.25	0.5	0.25	0.281
	Micafungin (6)	≤0.015	---	---	---	---

**Table 2:** Minimum inhibitory concentration (MIC) ranges, MIC_50_ and MIC_90_ values, modal MIC, and geometric mean (GM) MIC values for, isavuconazole, posaconazole, and voriconazole against *Aspergillus* section *Terrei* clinical isolates for which results were available for all three azoles against at least 10 isolates. MICs were measured after 48 h of incubation at 35 °C as the lowest concentration that resulted in 100% inhibition of growth. All values reported as mg/L. * GM *p*-value for posaconazole < 0.0001 vs. those for isavuconazole and voriconazole.

Species (No. Isolates)	Antifungal	MIC Range	MIC_50_	MIC_90_	Modal MIC	GM MIC
*Aspergillus* section *Terrei* (146)	Isavuconazole	0.125–2	0.5	1	0.5	0.514
	Posaconazole	≤0.03–1	0.06	0.25	≤0.03	0.087 *
	Voriconazole	0.125–4	0.5	1	0.5	0.463
*A. terreus* (100)	Isavuconazole	0.125–2	0.5	2	0.5	0.642
	Posaconazole	≤0.03–1	0.125	0.25	0.25	0.103 *
	Voriconazole	0.125–4	0.5	1	0.5	0.547
*A. citrinoterreus* (15)	Isavuconazole	0.125–1	0.25	1	0.25	0.301
	Posaconazole	≤0.03–0.25	≤0.03	0.25	≤0.03	0.055 *
	Voriconazole	0.125–1	0.25	0.5	0.25	0.287
*A. hortae* (19)	Isavuconazole	0.125–2	0.25	1	0.25	0.373
	Posaconazole	≤0.03–0.25	0.06	0.25	≤0.03	0.056 *
	Voriconazole	0.125–1	0.25	1	0.5	0.311

## Data Availability

Data will be made available to qualified researchers upon appropriate request.

## Supplementary Materials

The following supporting information can be downloaded at: https://www.mdpi.com/article/10.3390/pathogens12040579/s1, Figure S1: maximum likelihood trees showing phylogenies of individual loci; Table S1: site of cultures of clinical isolates; Table S2: percent matches of *A. pseudoalabamensis* type strain to other types within section *Terrei*.
