# Supplementary material for: Species Distribution and Antifungal Susceptibilities of Aspergillus Section Terrei Isolates in Clinical Samples from the United States and Description of Aspergillus pseudoalabamensis sp. nov
Source: Pathogens. 2023 Apr 11;12(4):579. doi: 10.3390/pathogens12040579 (PMC10142542; doi:10.3390/pathogens12040579)
Supplement: Supplementary file 1 [file pathogens-12-00579-s001.zip › pathogens-2260582-supplementary.pdf]

Supplemental Table S1. Site of culture of 278 *Aspergillus* section *Terrei* clinical isolates between October 2015 and January 2020 from across the U.S.

Culture Site	Percent
Lower respiratory tract	46.8%
Upper respiratory tract	22.7%
Lower extremity	7.2%
Upper extremity	2.9%
Back/abdomen	2.9%
Head	2.2%
Blood	1.8%
Eye	1.8%
Lung tissue	1.8%
Heart	1.4%
Bone	1.1%
Unknown	7.2%

Supplemental Table S2. Percent matches of *A. pseudoalabamensis* type strain to other types strains within *Aspergillus* section *Terrei* based on BLASTn searches. These matches are to Type strains, as indicated by the T superscript.

***A. pseudoalabamensis* UTHSCSA DI16-489 [T]**		
Region	Top Match	Percent Match
ITS	*A. heldtiae* PPRI 4229 [T]	99.51
	A. alabamensis CBS 125693 [T]	99.18
	A. terreus ATCC 1012 [T]	99.01
	A. hortae CBS 124230 [T]	99.01
BenA	*A. alabamensis* CBS 125693 [T]	95.36
	A. jilinensis CBS NN058401 [T]	94.79
	A. heldtiae PPRI 4229 [T]	92.89
	A. hortae CBS 124230 [T]	92.66
CaM	*A. heldtiae* PPRI 4229 [T]	96.17
	A. jilinensis CBS NN058401 [T]	96.12
	A. alabamensis CBS 125693 [T]	96.11
	A. citrinoterreus GM228 [T]	96.05
RPB2	*A. alabamensis* CBS 125693 [T]	98.53
	A. heldtiae PPRI 4229 [T]	97.80
	A. citrinoterreus GM228 [T]	97.80
	A. hortae CBS 124230 [T]	97.43

Supplemental Figure S1 Maximum likelihood trees showing phylogenies of the individual loci.

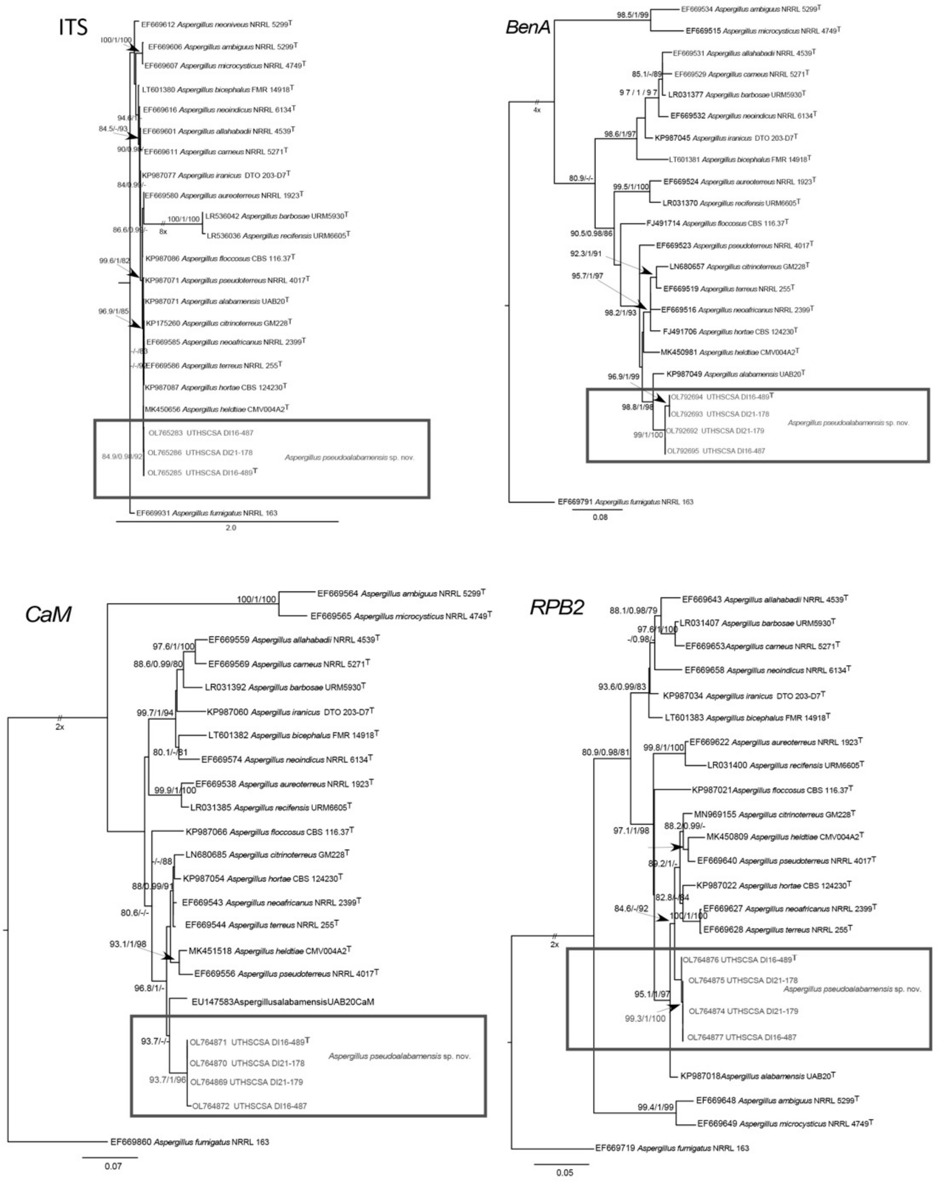

Figure S1. showing the placement of the new species of *Aspergillus* in the section *Terrei* and its relationship with other species in the section. Support values for SH-aLRT (≥ 80 %), aBayes (≥ 0.95), BS (≥ 75%) respectively are indicated on the nodes. T = represent type strains. GenBank accession numbers for all sequences are shown before the names and strain accession numbers are shown after the name. Dashes represent values lower than SH-aLRT (80%), aBayes (0.95), and BS (75%).
